# A Nerve Injury After Total Hip Arthroplasty from Etiology to Treatment: A Narrative Review

**DOI:** 10.3390/jcm15020563

**Published:** 2026-01-10

**Authors:** Alberto Di Martino, Matteo Brunello, Isabella Giannini, Manuele Morandi Guaitoli, Chiara Di Censo, Federico Pilla, Cesare Faldini

**Affiliations:** 1 I Orthopedic and Traumatology Department, IRCCS Istituto Ortopedico Rizzoli, 40136 Bologna, Italy; matteo.brunello@ior.it (M.B.); isabellasofia.giannini@ior.it (I.G.); manuele.morandiguaitoli@ior.it (M.M.G.); chiara.dicenso@ior.it (C.D.C.); federico.pilla@ior.it (F.P.); cesare.faldini@ior.it (C.F.); 2 Department of Biomedical and Neurimotor Science-DIBINEM, University of Bologna, 40136 Bologna, Italy

**Keywords:** total hip arthroplasty, nerve injury, surgical approach, bikini, sciatic nerve, femoral nerve

## Abstract

Total hip arthroplasty (THA) is a widely performed procedure that significantly enhances patients’ quality of life. However, nerve injury remains a concerning complication, with an incidence ranging from 0.6% to 3.7%, depending on patient and surgical variables. This narrative review provides a comprehensive overview of nerve injuries associated with THA, focusing on etiology, risk factors, clinical manifestations, prevention, and treatment strategies. The most affected nerves include the sciatic, femoral, lateral femoral cutaneous (LFCN), superior gluteal, and obturator nerves. Anatomical factors such as developmental hip dysplasia (DDH), limb length discrepancy, and aberrant nerve courses, along with patient-specific conditions like female sex, obesity, and pre-existing spinal disorders, increase the risk of nerve damage. Surgical complexity, revision procedures, and surgeon experience also influence injury likelihood. Clinical manifestations range from sensory disturbances to motor deficits including foot drop, Trendelenburg gait, or impaired knee extension, depending on the nerve involved. Diagnosis is primarily clinical, supported by electrophysiological studies and imaging when needed. Prevention hinges on careful preoperative planning, appropriate surgical approach selection, meticulous intraoperative technique, and attention to limb positioning. Treatment is typically conservative, involving pain control, physical therapy, and neurostimulation. In refractory or severe cases, interventions such as nerve decompression, repair, or tendon transfer may be considered. Pharmacological agents including vitamin B12, tacrolimus, and melatonin show potential in promoting nerve regeneration. Although most nerve injuries resolve spontaneously or with conservative measures, some cases may result in long-term deficits. Understanding the mechanisms, risk factors, and management strategies is essential to mitigating complications and optimizing functional outcomes in patients undergoing THA.

## 1. Introduction

Total hip arthroplasty (THA) is a transformative procedure that has profoundly improved the quality of life for patients with hip joint disorders, ranging from osteoarthritis to femoral head fractures [1,2]; THA offers effective relief from pain and a significant restoration of function. It is one of the most performed surgical interventions in orthopedics, accounting for over 1 million procedures performed worldwide each year [3,4]. With advancements in surgical techniques, implant design, and postoperative care, THA has become a reliable and long-lasting solution for individuals suffering from debilitating hip conditions, allowing them to regain mobility and to return to daily activities with minimal pain and discomfort [5].

THA can be performed through several surgical approaches, the most frequently used being posterolateral (PL), direct lateral (DL), and direct anterior (DAA) approaches. Each of these has distinct advantages and disadvantages, which influence both surgical outcomes and patients’ recovery. PL is the most commonly performed approach worldwide: it provides excellent exposure of the hip joint and preserves the hip abductors. However, it is associated with a higher risk of postoperative dislocation due to approach-specific factors. DL performance is associated with a lower dislocation rate, but it requires severance of the abductor muscles, which may result in a limp due to impaired hip function. A DAA uses the natural interval between the tensor fasciae latae and sartorius muscles to access the capsule and joint, providing not only negligible muscle damage but also faster recovery times and a quicker return to daily activities [6].

Nerve injury is a potential complication of all these approaches, occurring in 0.6% to 3.7% of patients [7]; in fact, nervous involvement at the femoral, sciatic, obturator, superior gluteal, and lateral femoral cutaneous nerve (LFCN) has emerged as a critical issue when dealing with the complications of THA [8]. The reported variability in incidence depends on anatomical variants, surgical technique, the type of surgery (primary or revision THA), and patient-related factors [9,10]. When these complications occur, motor deficits, functional compromise, persistent pain and numbness can overcome the benefits associated with THA surgery and affect overall patient satisfaction [11].

While numerous studies have compared the effectiveness of different surgical approaches used in THA [7], there is a notable deficiency in comprehensive reviews specifically focusing on the nerves at risk with each of these and on the strategies for preventing and treating associated injuries. Therefore, the primary aim of this study is to investigate nerve injuries—specifically the sciatic, femoral, lateral femoral cutaneous, obturator, and superior sciatic nerves—in the different approaches used for THA. Anatomical considerations that make these nerves susceptible to injury will be analyzed, providing a thorough overview from etiology to treatment strategies. By highlighting the principal aspects of nerve injuries, including risk factors, preventive measures, and therapeutic options, valuable insights will be furnished to enhance the overall safety and efficacy of THA surgery.

## 2. Incidence

The incidence of nerve injuries following THA varies based on the specific nerve involved and the surgical approach utilized. Among the most commonly affected nerves, the LFCN is particularly susceptible to injury, with reported rates ranging broadly from 2% to 81%, especially in cases performed through the DAA [12,13,14]. This variability arises from differences in surgical technique, patient demographics, and temporal and diagnostic criteria used for the diagnosis [14].

Femoral nerve (FN) injury, while less frequent [15], represents the overall second most common nerve involvement in THA surgery after the sciatic nerve (SN), with incidence rates between 0.01% and 2.3% [11]. Despite being reported anecdotally, all the approaches that insist on an anterior aspect of the tightness represent the main risk factors for intra- and postoperative FN compromise [11].

The superior gluteal nerve (SGN) can be affected during THA, albeit with a lower frequency, with incidence rates ranging from 0.3% to 1%; however, such lesions may be underreported because of the difficulty in diagnosis. The risk of injuring this nerve also depends on surgical approach and individual anatomical factors, with a notably higher risk associated with the direct anterior approach [16].

SN injury is among the most serious and prevalent complications of THA, accounting for 79% of all permanent neurological injuries after THA surgery. It is associated with arthroplasty performance through a posterior approach and with all surgeries that apply traction over the neural structure, including those requiring limb elongation over 3–4 cm and THAs in post-traumatic pelvis patients because of the altered anatomy and potential adherences to the main axis of the nerve. It shows incidence rates from 0.2% to 2.8%, being significantly more frequent in complex primary hip arthroplasties, such as those performed in patients with dysplastic hips, and in revision THA surgery [11,17].

Lastly, obturator nerve (ON) injuries are uncommon, with an incidence of approximately 0.5% to 1%. Although rare, an ON injury can considerably impact functional outcomes and patients’ quality of life. This low rate is partly due to the nerve’s relatively protected position within the pelvis, though injuries to this nerve can still have significant functional consequences (Table 1).

## 3. Clinical Presentation

Nerve injuries following THA present with a range of distinct clinical manifestations. Across all nerve injuries, symptoms highlight the vital role of these structures in maintaining normal motor and sensory functions, underscoring the need for early recognition and targeted management.

LFCN injuries commonly result in meralgia paresthetica, a sensory neuropathy characterized by numbness, tingling, burning pain, and varying degrees of hypoesthesia, dysesthesia, or hyperesthesia at the anterolateral thigh. These symptoms, often exacerbated by prolonged standing or walking, are not associated with motor deficits, and joint function is intact [12,18,19]. In contrast, FN injuries lead to quadriceps weakness, causing difficulty in knee extension and activities including leg lifting or straightening, with severe cases resulting in total loss of knee extension, gait disturbances, and impaired weight-bearing. Sensory symptoms may concur, with neuropathic pain, numbness, or hypoesthesia at the anterior thigh and medial leg [17,20,21].

SGN injuries are difficult to diagnose; these manifest as hip abductor weakness, leading to a Trendelenburg gait, in which the pelvis drops on the unaffected side during single-leg weight-bearing. This condition causes difficulty in lateral walking, normal walking, and climbing stairs due to compromised gluteus medius and minimus muscles [22,23].

Patients with SN lesions can exhibit motor deficits that most often manifest as footdrop; weakness in knee flexion due to hamstring involvement may concur. Most often, patients experience sensory disturbances including numbness, tingling, or burning pain at the posterior or lateral side of the thigh, leg, and foot. Symptoms depend on the severity of injury, ranging from mild sensory disturbances to complete motor dysfunction, and these may vary in timing, with some patients experiencing immediate postoperative deficits and others developing delayed dysfunction [24].

Finally, ON injuries are marked by weakness in hip adduction and sensory deficits along the medial thigh. Clinically, this results in compromised leg crossing, decreased stability during single-limb weight-bearing, and discomfort or instability while walking [25].

## 4. Risk Factors

Although a great portion of nerve palsies after THA are still of unknown etiology [11], there are certain conditions that are associated with an increased rate of complications. Risk factors can be categorized into patient-associated, whether clinical or anatomical, and surgery-associated (Table 2).

### 4.1. Clinical Factors

- Female gender is considered a risk factor for nerve injury after THA because of two principal reasons: First, females tend to have less soft tissue mass, predisposing them to nerve injury. Second, they are associated with a higher incidence of DDH [15,21,26]; in patients with DDH, the lower limb is often shortened before surgery and a significant elongation and offset modification is observed after THA. Additional factors that contribute to increasing the risk of nerve injury in females are a narrower pelvis and shorter stature, pregnancy, the presence of an insufficient or dysplastic hip, and previous pelvis or acetabular fractures [17,27,28].
- Patients below 50 years of age show a higher risk of nerve injury after THA compared with their older counterparts. The risk is approximately 7 times higher in younger patients; reasons include the longer average operative time because of the greater forces required to achieve an adequate surgical exposure with retractors. Furthermore, younger patients require THA because of secondary arthritis, encountered after DDH, Perthes’ disease, slipped capital femoral epiphysis, and post-traumatic arthritis in most cases.
- Obesity, which may alter the anatomical landmarks and increase the difficulty of the procedure, can also predispose patients to nerve injury [14,15,21,26].
- Patients under treatment with anticoagulant or platelet anti-aggregate treatment are susceptible to the formation of a psoas hematoma, which, in turn, may lead to compression of the FN. Some patients may develop postoperative hematomas because of an unrecognized coagulative disorder [29].
- The presence of a pre-existing spinal disease represents an independent factor increasing the risk of developing a nerve injury after THA [14,15,21,26]. The “double-crush” theory, according to which nerves become less tolerant to compression or stretching at a second anatomical site if they have pre-existing compression somewhere else along their course, determines an increased risk: classical examples include lumbar disc herniation and spinal stenosis [11], posing a question about the correct timing of surgery in patients affected by lumbar spine and hip diseases [30,31].

### 4.2. Anatomy-Associated Factors

**Figure 1:**
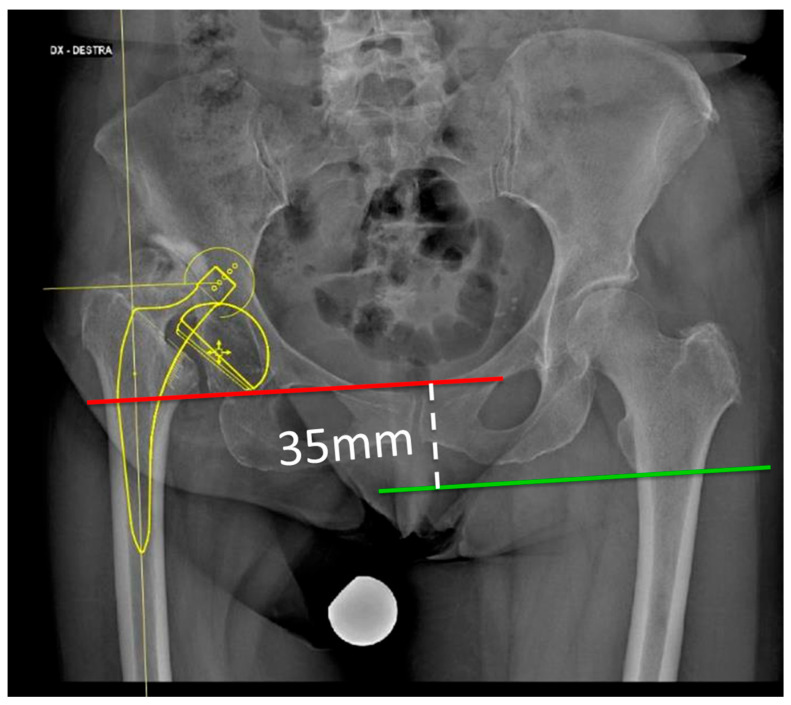
Severe DDH of the right hip, with an iliac dislocation and 35 mm of leg length discrepancy. This case represents an elevated nerve injury risk due to the traction needed to reduce the hip at the true acetabulum.

- Anatomical variations or severe deformities at the pelvis and hip can significantly increase the risk of nerve injury during THA procedures [25]. The proximity of the sciatic, femoral, and obturator nerves to the surgical field makes them vulnerable to damage, especially when there are deviations from the typical anatomy. Variations in the course, branching patterns, or location of these nerves can make them more susceptible to inadvertent traction, compression, or direct injury during the procedure by retractors. For instance, an unusually high or low bifurcation of the SN places it closer to the operative field, increasing the likelihood of intraoperative nerve entrapment or stretching. Similarly, the Fan-Type anatomical variant of LFCN is most prone to injury [32]; in fact, because of the fanning of the branches, damage to the nerve is almost unavoidable. Unfortunately, no routinary diagnostic tool allows altered anatomical patterns to be determined preoperatively; therefore, surgeons cannot anticipate potential challenges and adjust the surgical technique accordingly, minimizing the risk of nerve injury.
- Anatomical bone and soft tissue pathology, including DDH, is associated with 4 times higher odds of nerve injury; this factor could be due to the altered anatomy and nerve course but also to the increased surgical time and difficulty of the surgery. Similarly, patients affected by post-traumatic arthritis show 3.4 higher odds of nerve injury. Similarly, variations in the orientation or depth of the acetabulum can alter the exposure requirements, potentially leading to increased manipulation of close nerves.
- Limb length discrepancy, either preoperative or after THA surgery, is associated with increased risk of postoperative nerve injury. It is reported that an overall lengthening greater than 3–4 cm after THA increases the risk of nerve injury in the patient by 28%, the damage being caused by traction over the neural structures [33] (Figure 1).

### 4.3. Surgery-Associated Factors

**Figure 2:**
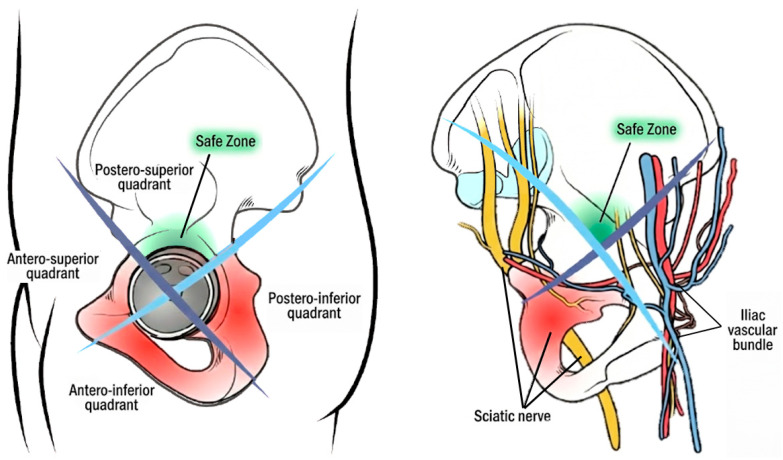
The figure illustrates the pelvis from both an external view (**left panel**) and an internal view (**right panel**) to demonstrate the anatomical relationships relevant to acetabular screw placement. The blue and light-blue lines are imaginary reference lines that divide the acetabulum into four quadrants—antero-superior, antero-inferior, postero-superior, and postero-inferior—all intersecting at the center of the acetabulum. The dark-blue line extends from the anterior superior iliac spine (ASIS) through the center of the fovea and beyond, while the light-blue line is drawn perpendicular to it. The green-shaded area indicates the safe zone, corresponding to the postero-superior quadrant, which is relatively free of critical neurovascular structures, whereas the remaining quadrants represent areas of increased risk. The internal view highlights the close contiguity of the iliac vascular bundle with the antero-superior quadrant and the proximity of the sciatic nerve within the antero-inferior quadrant.

- Experience of the surgeon: The operative volume of the operating surgeon influences the incidence of postoperative complications, including nerve injuries. It has been demonstrated that for every 50 THAs performed in the preceding year, there is a 13% decrease in the risk of injury. This may partially be explained by the shorter duration of surgery as the surgeon’s experience grows in the performance of both primary and revision THAs [10]. There are several factors related to surgeon inexperience that can increase the likelihood of neural injury developing. Among the most important are the following: improper retractor placement; improper patient positioning; excessive traction over the nerve; increased duration of surgery or the requirement of additional procedures including iliac harvest of bone grafting, implantation of wires, and screw fixation at the inner acetabulum; leakage of bone cement; and pelvic osteotomy performance [11,12,14,16,24,27,28].
- Revision total hip arthroplasty (rTHA): This is a significant risk factor for the development of a nerve injury compared to primary THA. The incidence of nerve injury in revision THA is considerably higher, with studies reporting rates as high as 7.6%, compared with 0.6–3.7% in primary procedures [12,16,17]. This increased risk is largely attributed to the complex surgical environment encountered during rTHAs, in which the presence of an altered local anatomy and scar tissue surrounding nerves, compromising their blood supply, make the nerves more vulnerable to traction [12,16,17]. Moreover, a larger postoperative hematoma is typically related to rTHA because of wider surgical exposure and soft tissue severance [8,24,28]. Additionally, these procedures usually last longer compared to primary THAs, and time is an independent risk factor for neural injury. Finally, the use of screws in atypical Wasielewski zones exposes the patient to an increased risk of neural injury because of the potential direct conflict with the tip of the screw [12,16,17], which is more common in revision cases compared to primary THAs [34] (Figure 2).

- Surgical approaches: Nerve injuries show a direct correlation with the surgical approach used in THA surgery; in fact, each approach is associated with particular nerve lesions because of the direct relationships encountered during the procedure.
  ○ Direct Anterior: The DAA is mainly associated with LFCN and FN lesions during THA surgery. FN injury can occur after the prolonged hip hyperextension required for the preparation of the femoral canal, because the nerve is particularly vulnerable in this position [11,15,17]. The mechanisms of injury may be due to nerve section, compression, and prolonged ischemia secondary to nerve retraction, leading to trauma, stretching, or compression. Less cumbersome, but frequent, are lesions of the LFCN. These can occur at different levels and through several mechanisms, including stretching, compression, laceration, involvement of scar tissue formation, or entrapment during suturing of the fascia at the end of surgery. DAA, especially with the bikini incision [35], puts the nerve at risk because of the proximity of the nerve to the surgical distance between the tensor fascia latae and sartorius muscles [12,13,14,18,19]. Rarely, SGN and SN lesions can also occur during the DAA: a lateral circumflex femoral artery injury could, directly and indirectly, lead to injury of the terminal branches of the SGN during DAA THA [16]. Moreover, especially in the anterior minimally invasive technique, SN injury may take place during trial and final hip reduction, as the nerve could possibly be compressed or strangled by the intact external hip rotators [11].
  ○ Direct Lateral: This approach is associated with the risk of injury to the FN in a manner similar to the DAA. Moreover, the DL approach is particularly associated with injury to the SGN, which innervates the hip abductors: lesions may occur if the gluteus medius is dissected 5 cm proximally to the greater trochanter; typically, the inferior branch is involved in up to 80% of cases. Nerve lesion may occur during gluteus medius splitting but also during muscle retraction to expose the acetabulum or during femoral elevation by retractors for implantation of the femoral stem.
  ○ Posterolateral: In the PL approach, the most commonly injured neural structure is the SN, accounting for 90% of all neural lesions with this approach. Due to its superficial course, which lies closer to the surgical wound, the common peroneal nerve component is at an increased risk of injury compared to the tibial nerve component. The common peroneal nerve consists of tightly packed fascicles, unlike the tibial nerve, and it has an abundance of connective tissue, which makes it more susceptible to transection and compression. There are several intraoperative or periprocedural causes that could harm the SN, including direct sharp or blunt trauma, compression by surgical clips, bony wires, direct compression or thermal lesion by cement extrusion, intraneural or perineural hematoma, vascular issues, and excessive leg lengthening [11]. However, in more than 50% of cases, the exact mechanism of injury cannot be identified.

## 5. Diagnosis

The event of a nerve injury requires a prompt diagnosis to improve the prognosis [15]. The diagnosis of a neural injury is primarily clinical, relying on the patient’s history and typical symptoms [25]. A detailed physical examination may reveal hypoesthesia or hyperesthesia at the lateral thigh with exacerbation of symptoms during provocative maneuvers, such as when extending the hip or applying pressure over the anterior superior iliac spine, suggesting an LFCN compromise [12,18,19]. Moreover, a neurological examination assesses quadriceps muscle strength and sensory function, paying attention to the patellar tendon reflex, to diagnose a lesion at the FN [17]. The clinical presentation can be highly suggestive, as in the case of SN injuries: patients may present with motor deficits, such as foot drop or weakness at knee flexion, together with sensory disturbances, particularly numbness or paresthesia at the posterior thigh, leg, and foot. Ankle reflexes may also be diminished or absent [24]. In the case of SGN or ON lesions, the Trendelenburg sign, the clinical marker of gluteus medius weakness, and evaluation of adductor muscle strength and sensory function at the medial thigh are the key for diagnosis. Electrophysiological studies, including nerve conduction studies and electromyography, can be employed to confirm the diagnosis, to assess the extent of the nerve damage, and to differentiate between neuropraxia, axonotmesis, and neurotmesis [20,23,24,36]. These are performed immediately after clinical suspicion; however, EMG testing typically reveals the entirety of the damage only 3 to 4 weeks after trauma. Imaging modalities such as ultrasound or magnetic resonance imaging (MRI) can be useful in ruling out other causes of neurological compromise, including hematomas and nerve compression (Table 3) [37].

## 6. Prevention

Preventing nerve injury during THA requires a combination of precise surgical techniques and awareness of anatomical variations:

- Surgical Approach Selection: Selecting the optimal surgical approach is crucial in minimizing the risk of SN injury. Surgeons should carefully evaluate the patient’s specific anatomy and risk factors to determine the most suitable approach. Preoperative imaging studies, such as MRI or CT scans, can help identify anatomical variations or abnormalities that may influence the choice of surgical approach, allowing for tailored strategies that reduce the likelihood of nerve damage [16,38]. The DAA is associated with higher incidence rates of the LFCN, FN, and SGN and its muscular branch entering the tensor fasciae latae due to their proximity to the surgical field [11,12,13,14,18,19,39]. For patients identified as high-risk for SN injury, the direct anterior or anterolateral approaches are often preferred because these typically involve less manipulation close to the SN compared to the PL approach. In patients suitable for the PL approach, it is essential to take additional precautions to protect the SN: these include precise dissection techniques to avoid unnecessary traction, including combined hip extension and knee flexion during exposure, minimizing the retraction required during surgery [8,24]. Depending on the patient’s anatomy and the complexity of the case, approaches like the DA or LA may offer better visibility and control, reducing the likelihood of nerve damage compared to the PL approach [25].
- Patient Positioning: Proper positioning of the patient on the operating table may contribute to a decrease in excessive traction, flexion, extension, rotation, or compression to critical regions (lateral thigh, ischial tuberosity, and fibular head) [21,24]. Tsurumi et al. [37], by analyzing the effects of leg position on femoral neurovascular bundle location using MRI, demonstrated that the external rotation and extension of the hip affects the femoral artery, vein, and nerve locations; these factors could decrease the risk of direct injury or traction on the nerves. Proper padding during patient positioning and careful alignment of the limbs can help avoid positioning-related nerve injuries. The patient should be positioned to keep neutral alignment of the hip joint; the surgical team should regularly check and adjust the patient’s position throughout the procedure to maintain optimal alignment [38]. Additionally, minimizing the time in which the patient stays in any position could further decrease the likelihood of prolonged nerve compression.
- Surgical Technique and Intraoperative Care: In THA surgery, the first precaution is careful incision performance. For example, in the DAA, it is important to keep the incision as minimal as possible, avoiding an extension in the zone of the LFCN course [39]: incision of the fascia over the belly of the tensor fascia latae muscle decreases the risk of direct nerve injury, as the nerve lies more medial and often runs alongside the sartorius muscle [40,41]. Furthermore, during surgery, gentle handling of tissues is required: meticulous retractor placement and avoiding prolonged traction and hyperextension on the hip joint are crucial to minimize compression or ischemia on the nerve [27,42]. It is important to avoid placing retractors or applying pressure near the anterior superior iliac spine (ASIS), which the LFCN lies close to [12]. Intraoperative monitoring, such as the use of electromyography (EMG), is not routinely performed, even though it can guide and provide real-time feedback on nerve integrity, allowing the surgical team to promptly address any signs of nerve compromise [22,23,38,42]. Ishimatsu et al. [20] demonstrated that 77% of the patients undergoing THA surgery with the DAA had reduced amplitude at the FN after the anterior retractor was placed on the anterior wall of the acetabulum, and the cause could be the retractor itself compressing the FN through the iliopsoas muscle bulk. Fortunately, all the patients showed complete strength preservation of knee extension without numbness of the FN in the postoperative period. Although the reduction in potential appeared clinically not significant, placement of the anterior retractor should be performed with careful attention to the FN. Similarly, the study by Satcher et al. [42], which used motor-evoked potential (MEP) monitoring, showed that hip flexion during posterior acetabular retraction was a cause of SN damage, demonstrating the importance of nerve isolation and proper retraction placement before distraction and implant placement. Moreover, cement positioning requires SN protection to decrease the risk of intraoperative damage [24].

## 7. Treatment

In the event of nerve injury during THA surgery, management focuses on both symptomatic relief and promotion of nerve recovery: it involves a combination of conservative and surgical interventions, depending on the severity of injury and the patient’s response to initial treatments.

Patient Education: Patients should be informed about the nature of their condition, the expected course of recovery, and strategies for managing symptoms. Understanding that most injuries have a good prognosis could help alleviate patient concerns and improve overall satisfaction after recovery [19].

Conservative Management: Most nerve injuries are managed conservatively [14,18,19]. Initial treatment should include the following:

- Pain Management: This is a critical component of the initial conservative approach of patients with nerve injury. Patients may experience significant neuropathic pain, which can be managed by a combination of nonsteroidal anti-inflammatory drugs (NSAIDs), gabapentinoids, or tricyclic antidepressants. These medications help to alleviate pain and improve patient comfort, promoting engagement in rehabilitation protocols [18].
- Physical Therapy: A structured physical therapy program is essential for promoting recovery and preventing long-term disability; it should begin as early as possible to prevent secondary complications such as joint stiffness, muscle atrophy, and loss of function. Therapy may include exercises to alleviate discomfort and to improve limb strength [18]; these focus on the strengthening of the quadriceps muscle to improve knee extension in the case of FN compromise, on the strengthening of gluteal muscles in the case of SGN lesion, or on empowerment of the calf or anterior tibialis muscle in the case of SN compromise [15,21]. Moreover, as the patient improves, therapy may be adjusted to include more advanced exercises aimed at restoring normal function and mobility [36]. Finally, techniques such as neuromuscular re-education and proprioceptive training are particularly beneficial in restoring function and preventing long-term deficits [43]. In this context, the use of muscle trans-cutaneous electrical stimulation is extremely important to promote nerve recovery. The duration of stimulation is recommended to be 3 to 6 months to obtain a clinical reduction in the pain [44].
- Follow-Up and Monitoring: Continuous follow-up is necessary to monitor patient recovery and to adapt treatment plans as needed. Regular neurological assessments (EMG and nerve conduction studies) and functional evaluations should be conducted to track improvements in muscle strength, gait, functional capacity, and sensory function or to detect any signs of ongoing nerve compression or deterioration [15,20,21,45].
- Pharmacological Therapy: Several experimental drugs showed promising results in enhancing peripheral nerve regeneration and in reducing scar formation. Hyaluronic acid (HA), a glycosaminoglycan, acts as an anti-adhesion agent by inhibiting lymphocyte and macrophage migration, reducing fibroblast proliferation and improving axonal growth, and it is often used in gel or film. Tacrolimus (FK506), an immunosuppressant, promotes axonal regeneration by binding to FKBP12/52, stimulating growth-associated protein GAP-43, and reducing fibroblast proliferation via apoptosis. Cyclosporin A shares similar immunosuppressive properties, inhibiting T-cell activation via calcineurin blockage, with additional anti-fibrotic effects. Melatonin, a pineal hormone, provides antioxidant and neuroprotective benefits by scavenging free radicals, enhancing Schwann cell proliferation, and potentially limiting neuroma formation. Methylprednisolone, a glucocorticoid, reduces inflammation by blocking cytokines like TNF-α and IL-1β, inhibiting macrophage recruitment and lipid peroxidation. Vitamin B12 (methylcobalamin) is crucial for myelin synthesis, axonal transport, Schwann cell proliferation, and DNA methylation, and it also exhibits antioxidant effects. Riluzole, a sodium channel blocker, protects neurons by preventing calcium influx and excitotoxicity, promoting neurite outgrowth. 4-Aminopyridine, a potassium channel blocker, restores action potentials in demyelinated axons, enhancing functional recovery. Verapamil, a calcium channel blocker, limits scar tissue by inhibiting fibroblast collagen production and inflammatory signaling, aiding nerve regeneration when applied topically [46].

**Figure 3:**
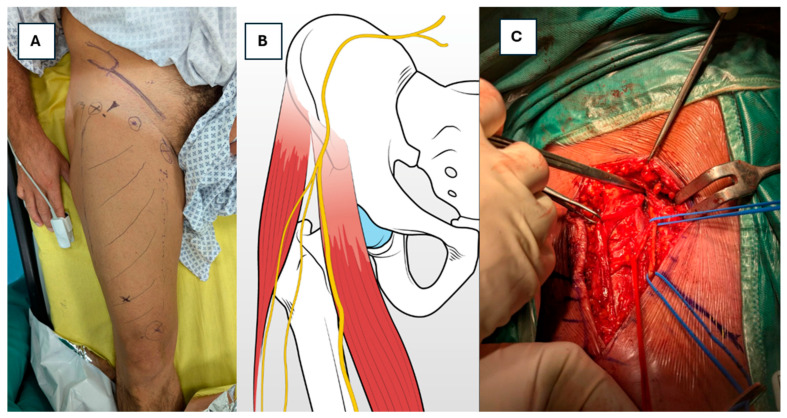
A 62-year-old patient who underwent a right THA 18 months previously at another institute and reported meralgia paresthetica not resolved with conservative treatment. (**A**) Patient-marked distribution of painful area following right THA—the location corresponds to LFCN sensory territory (anterolateral thigh). (**B**) Schematic anatomical relation of the LFCN to the ASIS and tensor fasciae latae. (**C**) Intraoperative photograph of complete LFCN release.

Interventions: If conservative management fails or symptoms persist beyond 6–12 months, consider advanced interventions:

- Nerve Blocks: Local nerve blocks can provide symptomatic relief from neuritic pain, and these are useful in distinguishing between sensory and motor nerve involvement [14,18].
- Surgical Exploration: In rare cases in which there is no improvement with conservative or less invasive treatments, surgical exploration may be necessary. This may involve decompression of the nerve in the case of entrapment or surgical repair if other structural issues are identified [14].
- Nerve Decompression: If diagnostic tests suggest that nerve injury is due to compression—either from scar tissue, hematoma, or other surgical complications—surgical nerve decompression may be necessary [21,36]. This procedure involves relieving the pressure on the nerve to restore its function and alleviate symptoms (Figure 3).

- Nerve Repair or Grafting: In the case of severe nerve damage, procedures like nerve repair or grafting may be required [24,36]. These surgical interventions are technically challenging and carry variable outcomes, with success rates depending on the extent of the nerve damage and on the timing of the intervention [36].
- Tendon Transfers: When conservative management does not yield sufficient recovery and chronic nerve damage is encountered, or when there are significant functional deficits that impair patients’ mobility and quality of life, tendon transfer procedures may be considered [21]. Most techniques were developed for poliomyelitis patients, and these can be applied to different districts. Tendons of the medial thigh, typically the adductor magnus [47], are transferred to contribute to knee extension, crucial for walking and weight-bearing activities. With functional surgery, at 6–12 months after surgery, the patient’s knee extension is partly restored, and ambulation is significantly improved [47]. Another frequent transfer is the posterior tibialis tendon for anterior tibialis, to contrast footdrop.

The decision to proceed with interventions should be made after thorough evaluation and consultation with a multidisciplinary team, including orthopedic surgeons, neurologists, and rehabilitation specialists [24]. For compressive lesions causing severe motor deficit or progressive neurological deterioration, urgent surgical decompression is recommended (days to a few weeks). For non-compressive injuries or minor nerve involvement (such as LFCN), observation with EMG follow-up is reasonable, considering decompression/exploration if there is no clinical or electrophysiological improvement by 6–18 months, depending on the severity of the injury and the nerve involved.

Postoperative care following advanced interventions often includes continued physical therapy and pain management, tailored to the patient’s recovery needs.

## 8. Prognosis

The prognosis of nerve injuries following THA depends on the type and severity of damage, with outcomes often favorable after timely and appropriate intervention, although some patients may experience residual deficits requiring long-term management and the use of assistive devices. LFCN injuries generally resolve favorably, with 96% of patients undergoing THA via the direct anterior approach experiencing spontaneous resolution of symptoms within three to six months [12,19]. Conservative management typically provides significant relief, and only 10% of patients report symptoms eight years after surgery [18]. Recovery is influenced by the extent of the initial injury, with partial injuries having better outcomes compared with complete transections. Symptoms such as hypoesthesia are associated with a worse prognosis, while dysesthesia often resolves spontaneously [19]. Persistent sensory disturbances are rare and usually mild, having little impact on daily activities or quality of life [12,18,19,39]. For FN injuries, the prognosis is also generally positive. Most patients experience significant improvement within six months to one year, regaining strength and function [11]. Residual weakness or sensory changes may persist in cases involving retraction-related neuropathy [15,17,26], but long-term outcomes are favorable with continued care and rehabilitation. Personalized follow-up plans help address any lingering effects and optimize recovery [26]. The SN, being more vulnerable to severe damage, presents a variable prognosis depending on the nature of the injury. Neuropraxia, involving temporary disruption without structural damage, has a favorable outlook, with near-complete recovery expected within weeks to months. However, more severe forms of injury, such as axonotmesis or neurotmesis, may lead to permanent functional deficits, requiring assistive devices or orthoses to manage limitations like foot drop [36]. Regular electrophysiological monitoring is essential for tracking recovery and guiding treatment adjustments [24,36,42]. SGN injuries generally have a positive prognosis, with many patients achieving significant improvement in muscle function and gait abnormalities through conservative management. Recovery can be gradual, and some individuals may experience residual weakness or balance issues despite thorough rehabilitation efforts. The outcome is heavily influenced by the severity of the injury and the timeliness and effectiveness of the intervention. Finally, ON injuries, though rare, present a variable prognosis. Most patients respond well to early conservative treatment, regaining function and experiencing alleviated symptoms. However, residual weakness or sensory deficits can persist in severe cases, depending on the extent of the injury and on the presence of complicating factors. The success of recovery also relies on effective rehabilitation and patient compliance with treatment protocols [43]. Overall, while nerve injuries following THA can significantly impact recovery, most patients achieve substantial improvements with appropriate interventions, allowing them to regain mobility and quality of life (Table 4).

## 9. Conclusions

Nerve injury following total hip arthroplasty remains an uncommon but potentially severe complication with relevant functional and psychosocial consequences. Although the overall incidence is relatively low, particularly in primary procedures, the impact on patient quality of life can be substantial when nerve damage occurs. The severity and prognosis of nerve injury are strongly influenced by the underlying mechanism, the extent of axonal involvement, and the timeliness of diagnosis and intervention. Careful preoperative assessment, including identification of established risk factors such as developmental dysplasia of the hip, younger patient age, pre-existing spinal pathology, and prior hip surgery remains essential to mitigate risk, particularly in anatomically complex cases. When postoperative neurological deficits are suspected, structured and timely monitoring is critical. Early and repeated clinical examinations should be combined with appropriately timed electrophysiological studies to assess the degree of nerve injury and to distinguish transient neuropraxia from more severe axonal damage. Imaging modalities play a key role in identifying potentially reversible causes, such as compressive hematomas or mechanical entrapment, that may require intervention. Prognosis is closely related to the severity of nerve damage and to the interval between injury and treatment, with earlier recognition and management offering a higher likelihood of meaningful neurological recovery. Compressive or progressive lesions warrant prompt surgical exploration, whereas non-compressive injuries require close surveillance and reassessment to guide decision-making. Transparent and ongoing communication between the surgeon and patient is essential to explain the nature of the injury, expected recovery timelines, and realistic functional outcomes. A structured, multidisciplinary approach to diagnosis and follow-up represents the most effective strategy to optimize recovery and minimize the long-term consequences of nerve injury after total hip arthroplasty.

## Figures and Tables

**Table 1:** Neural injury related to THA: incidence, surgical approach, and clinical complications.

Involved Nerve	Surgical Approach			Clinical Complications
	DAA	DL	PL	
**Lateral femoral cutaneous nerve** [12,13,14]	2–81%	Low	Rare	Meralgia paresthetica
**Femoral nerve** [15]	0.01–2.3%	0.01–2.3%	Rare	Quadriceps weakness, anterior thigh pain
**Superior gluteal nerve** [16]	0.3–1%	0.3–1%	Rare	Trendelenburg gait, abductor weakness
**Sciatic nerve** [11,17]	Low	Low-to-Moderate	0.2–2.8%	Foot drop, posterior leg paresthesia
**Obturator nerve**	Rare	Rare	Rare	Weakness in adduction, gait instability

**Table 2:** Summary table of the main clinical, anatomical, and surgery-related risk factors for nerve injury after total hip arthroplasty, indicating their approximate magnitude and relative impact. When precise quantitative data were unavailable, the degree of risk was categorized qualitatively based on published evidence and clinical relevance.

Category	Risk Factor	Approximate Proportion/Reported Measure	Relative Impact
**Clinical factors**	Female sex	Increased incidence compared to males	Low
	Age < 50 years	≈7-fold higher risk	High
	Obesity	Increased technical difficulty	Moderate
	Pre-existing spinal disease (double-crush phenomenon)	Qualitative increase	Moderate
**Anatomy-associated factors**	Developmental dysplasia of the hip (DDH)	≈4-fold increased odds	High
	Post-traumatic arthritis	≈3.4-fold increased odds	High
	Limb lengthening > 3–4 cm	≈28% increased risk	Moderate–High
	Aberrant nerve course/anatomical variations	Not quantifiable	Moderate
**Surgery-associated factors**	Revision total hip arthroplasty	Incidence up to 7.6%	High
	Prolonged surgical time	Qualitative increase	Moderate
	Low surgeon volume	Risk reduction ≈ 13% for every 50 THAs/year increase	Moderate
	Surgical approach	Variable according to nerve at risk	Low–Moderate

**Table 3:** Summary of the diagnostic approach to nerve injury after total hip arthroplasty, distinguishing between clinical examination, electrophysiological assessment, and imaging modalities for each nerve involved.

Nerve Involved	Main Clinical Features	Clinical Examination (Bedside Assessment)	Electrophysiological Studies (NCS/EMG)	Imaging and Additional Tests
**Lateral femoral cutaneous nerve (LFCN)**	Paresthesia, numbness, or burning pain over the anterolateral thigh (meralgia paresthetica)	Sensory mapping of the anterolateral thigh; palpation near the ASIS; symptom reproduction with hip extension	Often normal; may support diagnosis in selected cases	High-resolution ultrasound to assess entrapment or scar tissue
**Femoral nerve**	Quadriceps weakness, reduced or absent patellar reflex, anterior thigh sensory loss	Manual muscle testing of knee extension; patellar reflex evaluation	NCS/EMG to confirm injury and assess axonal loss (optimal ≥3–4 weeks after injury)	MRI or ultrasound to exclude psoas or iliacus hematoma
**Sciatic nerve**	Foot drop, posterior leg pain or sensory loss, weakness of ankle dorsiflexion or plantarflexion	Strength testing of ankle and toe movements; sensory examination; gait assessment	NCS/EMG for localization and severity assessment	MRI or ultrasound to identify compressive lesions or entrapment
**Superior gluteal nerve**	Hip abductor weakness, Trendelenburg gait	Trendelenburg test; hip abductor strength testing	EMG of gluteus medius and minimus muscles	MRI to evaluate muscle denervation or atrophy
**Obturator nerve**	Medial thigh sensory deficit, adductor muscle weakness	Manual testing of hip adduction; gait analysis	EMG of adductor muscles	MRI or ultrasound if compression is suspected

**Table 4:** Main treatments for neural injury after THA.

Treatment	Clinical Indication	Prognosis/Expected Outcome
Conservative Management	First-line option for most nerve injuries	High success in mild cases; symptoms often resolve within 3–6 months (especially LFCN)
Targeted Physical Therapy	Muscle strengthening, mobility recovery, prevention of contractures	Improved functional outcomes with adherence; tailored to nerve affected
Neuroregenerative Pharmacotherapy	Support axonal regeneration and reduce fibrosis (e.g., tacrolimus, B12)	Promising results in preclinical and early clinical studies
Neuromuscular Stimulation	Enhance muscle reinnervation, reduce pain	Moderate improvement in strength and pain over 3–6 months
Interventional Pain Management/Decompression	Persistent pain or compression; failure of conservative treatment > 12 months	Good prognosis with early surgical decompression
Surgical Repair/Grafting/Tendon Transfers	Severe axonal loss, irreversible deficits	Variable outcomes: tendon transfer may restore key functions (e.g., knee extension, foot dorsiflexion)

## Data Availability

No datasets were generated or analyzed during the current study.

## Abbreviations

The following abbreviations are used in this manuscript:

THA	Total Hip Arthroplasty
LFCN	Lateral Femoral Cutaneous Nerve
DAA	Direct Anterior Approach
DL	Direct Lateral Approach
PL	Posterolateral Approach
FN	Femoral Nerve
SGN	Superior Gluteal Nerve
SN	Sciatic Nerve
rTHA	Revision Total Hip Arthroplasty
DDH	Developmental Dysplasia of the Hip
ASIS	Anterior Superior Iliac Spine
EMG	Electromyography
NCS	Nerve Conduction Studies
MEP	Motor-Evoked Potential
NSAIDs	Nonsteroidal Anti-Inflammatory Drugs
HA	Hyaluronic Acid
GAP-43	Growth-Associated Protein 43
TNF-α	Tumor Necrosis Factor-alpha
IL-1β	Interleukin-1 beta
